# Supplementary material for: Content-rich biological network constructed by mining PubMed abstracts
Source: BMC Bioinformatics. 2004 Oct 8;5:147. doi: 10.1186/1471-2105-5-147 (PMC528731; doi:10.1186/1471-2105-5-147)
Supplement: Additional File 2 — The original results of the above study (non-essential files are deleted to keep the file size under the limit set by BMC bioinformatics). [file 1471-2105-5-147-S2.bz2 › chilibotAdditionalFile2/dip40/725ID9822594E18074/html/HLYD_HLYB.html]

 


 **HLYD** and **HLYB** 
  
Found 77 abstracts in PubMed, retrieved 40.  
 

 What does Google say? 
 PDF only 
| .edu only 

---

**Interactive relationship** (e.g. stimulation, inhibition, etc)

**Neutral relationship**- This suggests that the presence of  **HlyB**  induces a structural change in  **HlyD** .  Ref: 10071218 Mol Gen Genet, 1999
- These results are consistent with an interaction between  **HlyD**  TolC and  **HlyB**   **HlyD** .  Ref: 10071218 Mol Gen Genet, 1999
- and b the C terminal sequence of  **HlyD**  interacts with periplasmic loop s of  **HlyB**  to form a closed channel spanning the periplasm.  Ref: 9393456 Mol Gen Genet, 1997

**Non-interactive relationship** (e.g. studied together, co-existance, homology, etc.)

- This PagC HlyAs fusion protein was expressed and efficiently secreted into the culture supernatant by E. coli upon complementation with the hemolysin secretion proteins  **HlyB**  and  **HlyD** .  Ref: 8766698 Appl Microbiol Biotechnol, 1996
- **HlyB**  and  **HlyD**  are involved in secretion of the toxin.  Ref: 9521785 Biochemistry, 1998
- **HlyD**  and  **HlyB**  assembled a stable IM complex in the absence of TolC and substrate.  Ref: 9822594 EMBO J, 1998
- Identification of p60 antibodies in human sera and presentation of this listerial antigen on the surface of attenuated salmonellae by the  **HlyB**   **HlyD**  secretion system.  Ref: 1452342 Infect Immun, 1992
- A derivative of  **HlyD** , HlyD22, lacking the 40 N terminal residues of  **HlyD**  assembles into the inner membrane displaying the same stability with and without  **HlyB**  as wild type  **HlyD**  does.  Ref: 10071218 Mol Gen Genet, 1999
- Together with the inner membrane protein  **HlyD**  and the outer membrane protein TolC,  **HlyB**  is responsible for transport of the 107 kDa toxin HlyA from the cytoplasm, across both membranes of the cell envelope of Escherichia coli, directly to the medium.  Ref: 8366127 J Biol Chem, 1993
- Following the insertion between the reporter gene and hlyAs of a linker sequence that contains the information for potential cleavage sites for the outer membrane protease OmpT, two different fusion proteins PhoA HlyAs and CE HlyAs were shown to be cleaved by OmpT between the two parts during  **HlyB**   **HlyD**  mediated secretion.  Ref: 1603076 Mol Gen Genet, 1992
- The expression of a fully functional haemolysin translocation system comprising HlyC, A, B and D increases the sensitivity of E. coli to vancomycin 2.5 fold, compared with cells expressing  **HlyB**  and  **HlyD**  alone.  Ref: 7808392 Mol Gen Genet, 1994
- This sequence coded for the RTX secretion proteins ApxIIIB and ApxIIID, which showed 86 and 63% similarity to ApxIB and ApxID, respectively, and 83 and 63% similarity to  **HlyB**  and  **HlyD**  of Escherichia coli, respectively.  Ref: 7927703 Infect Immun, 1994
- Using a dual plasmid complementation system, we show that the alpha hemolysin  **hlyB**  and  **hlyD**  transport determinants are sufficient to allow secretion and activation of a functional metalloprotease species from E. coli, as are the comparable protease secretion functions of Erwinia chrysanthemi.  Ref: 1551853 J Bacteriol, 1992
- The export of Escherichia coli hemolysin across the cytoplasmic and the outer membranes requires the COOH terminal signal sequence of HlyA, the two specific translocator proteins  **HlyB**  and  **HlyD** , and the outer membrane protein TolC.  Ref: 9630987 Nat Biotechnol, 1996
- In the absence of  **HlyB** ,  **HlyD**  is also unstable and specific degradation products are detected, which co fractionate with the inner membrane, indicating in this case limited cleavage at specific sites.  Ref: 10071218 Mol Gen Genet, 1999
- Extracellular secretion of HlyA by the TetC  **HlyB**  hybrid required  **HlyD**  and TolC.  Ref: 1400227 J Bacteriol, 1992
- A 10.2 kb fragment that encoded hemolytic activity in the phage lysate was aligned by Southern blot hybridization to genes hlyC, hlyA,  **hlyB** , and  **hlyD**  of the Escherichia coli hemolysin operon, and expression of the A. pleuropneumoniae genes in E. coli revealed that they have the same functions as their E. coli analogs hlyIC encodes a protein that activates inactive 105 kDa prohemolysin I encoded by hlyIA to active hemolysin I, while hlyIB and hlyID are necessary for HlyIA secretion.  Ref: 1639473 Infect Immun, 1992
- This complex comprises a traffic ATPase  **HlyB**  and the 478 residue adaptor protein  **HlyD** , which contacts TolC during recruitment.  Ref: 11676535 J Mol Biol, 2001
- Selection for transport competence of C terminal polypeptides derived from Escherichia coli hemolysin the shortest peptide capable of autonomous  **HlyB**   **HlyD**  dependent secretion comprises the C terminal 62 amino acids of HlyA.  Ref: 7531275 Mol Gen Genet, 1994
- The genetic determinants for a fusion of its B subunit StxB, which mediates toxin binding to target cells, with the COOH terminus of Escherichia coli hemolysin A Stx HlyA has been combined with the determinants of the accessory translocator proteins  **HlyB**  and  **HlyD**  in a Notl cassette.  Ref: 8905616 Microb Pathog, 1996
- We have developed an export cloning system that is composed of two vectors one in which the fusion of the desired gene with the 3 end of hlyA is generated, and a second in which the sequences containing the fusion are combined with the accessory genes  **hlyB**  and  **hlyD** , thereby reconstructing the natural organization of the hly locus.  Ref: 9630987 Nat Biotechnol, 1996
- A recombinant r Salmonella typhimurium aroA vaccine strain was constructed which secretes the naturally somatic protein of Listeria monocytogenes, superoxide dismutase SOD, by the  **HlyB**   **HlyD**  TolC export machinery.  Ref: 9119463 Infect Immun, 1997
- Part about 30% of the expressed fusion protein CE HlyAs was secreted in E. coli carrying  **hlyB**  and  **hlyD**  genes.  Ref: 1603076 Mol Gen Genet, 1992
- The resulting 53 kDa hybrid protein was specifically secreted to the external medium in the presence of the haemolysin translocator proteins,  **HlyB**  and  **HlyD** .  Ref: 7500946 Mol Gen Genet, 1995
- The  **HlyB**   **HlyD**  dependent secretion of toxins by gram negative bacteria.  Ref: 1419114 FEMS Microbiol Immunol, 1992
- The toxin HlyA is exported from Escherichia coli, without a periplasmic intermediate, by a type I system comprising an energized inner membrane IM translocase of two proteins,  **HlyD**  and the traffic ATPase  **HlyB** , and the outer membrane OM porin like TolC.  Ref: 9822594 EMBO J, 1998
- these were termed EHEC  **hlyB**  and EHEC  **hlyD** .  Ref: 8936317 Microbiology, 1996
- These data suggest that the  **HlyB**   **HlyD**  TolC based antigen delivery system with attenuated r S. typhimurium as carrier is capable of inducing an immune response against mycobacterial antigens.  Ref: 10727883 FEMS Immunol Med Microbiol, 2000
- Production of active Serratia marcescens metalloprotease from Escherichia coli by alpha hemolysin  **HlyB**  and  **HlyD** .  Ref: 1551853 J Bacteriol, 1992
- In addition, we report on our r Salmonella typhimurium expression system combined with the  **HlyB**   **HlyD**  TolC export machinery for delivering the prominent mycobacterial antigen Ag85B for immune recognition.  Ref: 10076914 FEMS Immunol Med Microbiol, 1999
- Moreover, they suggest that the  **HlyB**   **HlyD**  TolC based antigen delivery system with attenuated Salmonella spp.  Ref: 9119463 Infect Immun, 1997
- Our mutational analyses of the HlyA targetting signal and definition for the first time of stages and intermediates in the  **HlyB**   **HlyD**  dependent translocation allow a discussion of the hemolysin export process in the wider context of protein translocation.  Ref: 1419114 FEMS Microbiol Immunol, 1992
- An Escherichia coli strain with wild type hemolysin secretion genes  **hlyB**  and  **hlyD**  and containing a cloned frpA gene secreted FrpA.  Ref: 8514394 Infect Immun, 1993
- Our data suggest that all beta Gal HlyAs fusion proteins generated are secreted, albeit with varying efficiencies, by the  **HlyB**   **HlyD**  TolC hemolysin secretion machinery under Sec proficient conditions.  Ref: 8842146 Mol Gen Genet, 1996
- The system which consists of the two specific membrane proteins  **HlyB**  and  **HlyD**  and the outer membrane protein TolC, recognizes on HlyA a C terminally located signal sequence of about 60 amino acids.  Ref: 9382730 Behring Inst Mitt, 1997
- Secretion of HlyA occurs without a periplasmic intermediate and is directed by an uncleaved C terminal targetting signal and the  **HlyB**  and  **HlyD**  translocator proteins, the former being a member of a transporter superfamily central to import and export of a wide range of substrates by prokaryotic and eukaryotic cells.  Ref: 1365905 Bioessays, 1992
- Our data suggest that TnhlyAs generated hemolysin fusion proteins are secreted efficiently by the  **HlyB**   **HlyD**  TolC hemolysin secretion machinery.  Ref: 9711856 FEMS Microbiol Lett, 1998
- The secretion of the 107 kDa hemolysin A HlyA from Escherichia coli is mediated by membrane proteins hemolysin B  **HlyB**  and hemolysin D  **HlyD** .  Ref: 7703231 Biochemistry, 1995
- However, hemolytic activity was found in the broth culture supernatant after transforming EDL 933 with the recombinant plasmid pRSC6 carrying the  **hlyB**  and  **hlyD**  genes from the E. coli alpha hemolysin operon.  Ref: 7868227 Infect Immun, 1995
- Secretion of Escherichia coli hemolysin is mediated by a sec independent pathway which requires the products of at least three genes,  **hlyB** ,  **hlyD**  and tolC.  Ref: 7816028 Mol Gen Genet, 1994
- This recombinant plasmid expressed a fusion protein Gcp F which was secreted into the culture supernatant by E. coli cells when the alpha hemolysin secretion functions  **HlyB**  and  **HlyD**  are supplied in trans.  Ref: 8150268 FEMS Microbiol Lett, 1994
- We show that an scFv HlyA hybrid secreted by Hly type I transporter TolC,  **HlyB** ,  **HlyD**  is accumulated in the extracellular medium with the disulphide bonds correctly formed.  Ref: 11309117 Mol Microbiol, 2001
- A recombinant r Salmonella typhimurium aroA vaccine that secretes the naturally secreted protein of Mycobacterium bovis strain BCG, Ag85B, by means of the  **HlyB**   **HlyD**  TolC export machinery termed p30 in the following was constructed.  Ref: 10727883 FEMS Immunol Med Microbiol, 2000
- In addition,  **HlyB**  itself appears to be less stable in the absence of  **HlyD** .  Ref: 10071218 Mol Gen Genet, 1999
- It is directed by an uncleaved C terminal targetting signal and the  **HlyD**  and  **HlyB**  translocator proteins, the latter of which are members of a transporter superfamily central to import and export of a wide range of substrates by prokaryotic and eukaryotic cells.  Ref: 1419114 FEMS Microbiol Immunol, 1992
- On the contrary, in the absence of both  **HlyB**  and TolC the half life of  **HlyD**  is approximately 110 min.  Ref: 10071218 Mol Gen Genet, 1999
- We engineered plasmid pETR14, containing the  **hlyB**  and  **hlyD**  genes of the Escherichia coli hemolysin operon, to express a fusion protein containing 720 amino acid residues from the nontoxic, receptor binding, carboxy terminus of C. difficile toxin A and the secretion signal of E. coli hemolysin A.  Ref: 9199470 Infect Immun, 1997
- In addition, we found that ApxIII could be secreted from E. coli by the heterologous RTX transporter proteins  **HlyB**  and  **HlyD** .  Ref: 8432615 Infect Immun, 1993
- The structure and function of the components of the HlyA secretion apparatus,  **HlyB** ,  **HlyD**  and TolC, have been studied in great detail.  Ref: 11755084 Trends Microbiol, 2002
- Second site suppressor mutations in  **hlyD**  and  **hlyB**  were obtained, which abolish the hypersensitive phenotype caused by the 3 terminal mutations in  **hlyD** .  Ref: 9393456 Mol Gen Genet, 1997
- A LacZ HlyAs, fusion protein carrying, at the C terminus of LacZ, the 60 amino acid signal sequence of HlyA was not secreted in the presence of  **HlyB**   **HlyD** .  Ref: 1552901 Mol Gen Genet, 1992
- To this end, Salmonella typhimurium SL7207 was used as carrier for secreted p60 HlyA fusion protein by Escherichia coli  **HlyB**  and  **HlyD**  transport proteins.  Ref: 7729919 Infect Immun, 1995
- In this way, mutations in the known secretion genes,  **hlyB** ,  **hlyD**  and tolC, were obtained.  Ref: 8437516 Mol Microbiol, 1993
- This result shows that in the presence of  **HlyB**  removal of TolC renders  **HlyD**  more unstable than it is in the absence of both  **HlyB**  and TolC.  Ref: 10071218 Mol Gen Genet, 1999
